# Первый российский регистр гипопаратиреоза с системой поддержки принятия врачебных решений

**DOI:** 10.14341/probl12796

**Published:** 2021-08-06

**Authors:** Е. В. Ковалева, А. К. Еремкина, А. Р. Айнетдинова, А. П. Милютина, Н. Г. Мокрышева

**Affiliations:** Национальный медицинский исследовательский центр эндокринологии; Национальный медицинский исследовательский центр эндокринологии; Национальный медицинский исследовательский центр эндокринологии; Национальный медицинский исследовательский центр эндокринологии; Национальный медицинский исследовательский центр эндокринологии

**Keywords:** гипопаратиреоз, регистр, методология

## Abstract

Хронический гипопаратиреоз относится к относительно редким заболеваниям, характеризующимся низким уровнем кальция крови и отсутствием или недостатком паратиреоидного гормона. Хроническое течение заболевания связано с необходимостью применения многокомпонентной лекарственной терапии, тщательного динамического мониторинга для снижения рисков развития осложнений заболевания, а также инвалидизации и смертности.С целью оценки фактической распространенности, заболеваемости гипопаратиреозом, анализа клинических особенностей и ключевых эпидемиологических характеристик заболевания в Российской Федерации в 2020 г. под эгидой ФГБУ «НМИЦ эндокринологии» Минздрава России разработан Всероссийский регистр пациентов с хроническим послеоперационным и нехирургическим гипопаратиреозом.В данной статье анонсируются основные цели и задачи данного проекта, методология ведения регистра хронического послеоперационного и нехирургического гипопаратиреоза, аналитические возможности его использования, в том числе инновационные разработки по внедрению системы поддержки принятия решений, призванные помочь специалистам в условиях реальной клинической практики следовать алгоритмам надлежащей диагностики и лечения заболевания, утвержденным клиническими рекомендациями по проблеме.Регистр хронического послеоперационного и нехирургического гипопаратиреоза расположен на единой платформе регистров эндокринопатий ФГБУ «НМИЦ эндокринологии» Минздрава России (http://gipopt.clin-reg.ru/).

## Abstract (en)

According to available research, chronic hypoparathyroidism is a relatively rare disease characterized by low serum calcium levels and the absence or deficiency of parathyroid hormone. The chronic course of the disease is associated with the multicomponent medical therapy, careful dynamic monitoring to reduce the risks of various complications in different organs and systems as well as disability and mortality.The Russian registry of patients with chronic postsurgical and nonsurgical hypoparathyroidism has started its work in 2020, based on data of the Endocrinology Research Centre. The main goals of the Registry are the assessment of the actual prevalence, incidence of hypoparathyroidism, the key epidemiological characteristics, the analysis of the clinical features and medical therapy of chronic hypoparathyroidism in Russian Federation.This article covers all objectives of this project, the methodology for maintaining the registry of chronic postsurgical and nonsurgical hypoparathyroidism, the analytical possibilities of its use, including the integration of a decision support system designed to help specialists in real clinical practice follow the algorithms for diagnosis and treatment of the disease, approved by clinical guidelines.The registry of chronic postsurgical and nonsurgical hypoparathyroidism is located on a single platform for the registers of endocrinopathies, regulated by the Endocrinology Research Centre (http://gipopt.clin-reg.ru/).

## ВВЕДЕНИЕ

Гипопаратиреоз является заболеванием, характеризующимся сниженной продукцией паратиреоидного гормона (ПТГ) околощитовидными железами (ОЩЖ), что приводит к нарушениям фосфорно-кальциевого обмена.

Основные симптомы гипопаратиреоза — гипокальциемия и связанные с ней нарушения: судороги, парестезии, нарушения сердечного ритма, ларинго-/бронхоспазм. Длительное течение заболевания, особенно в случае отсутствия его компенсации, приводит к развитию множественных осложнений, таких как поражение почек (нефролитиаз/нефрокальциноз), кальцификация мягких тканей, психоневрологические расстройства и др.

Наиболее частая причина заболевания — повреждение или удаление ОЩЖ при различных хирургических вмешательствах в области шеи с развитием послеоперационного гипопаратиреоза. В детском возрасте чаще встречается аутоиммунное поражение ОЩЖ в рамках аутоиммунного полигландулярного синдрома 1 типа (АПС 1 типа) — тяжелого заболевания с полигландулярной недостаточностью, требующего пожизненного проведения многокомпонентной заместительной терапии.

Лечение гипопаратиреоза включает назначение активных метаболитов/аналогов витамина D (альфакальцидола/кальцитриола) и солей кальция [1][2]. Патогенетическая терапия рекомбинантным человеческим ПТГ (рчПТГ) в настоящее время в Российской Федерации (РФ) практически не используется, поскольку препаратов рчПТГ по показанию гипопаратиреоза в РФ не зарегистрировано. Лечение рчПТГ может быть назначено по решению врачебной комиссии, как правило, только в специализированных центрах.

Нозологию принято относить к редким эндокринным заболеваниям, однако сведения о частоте гипопаратиреоза ограничены, и она может недооцениваться. По данным национального регистра Дании, распространенность гипопаратиреоза составляет в среднем 25 случаев на 100 тыс. населения [3], в Шотландии — 40 случаев на 100 тыс. [4], в США — 37 на 100 тыс. [5], в Норвегии — 10 случаев на 100 тыс. населения [6]. В РФ эпидемиологические источники по данной патологии отсутствуют, что, наряду с высокими рисками инвалидизации при отсутствии должного контроля над заболеванием, определило необходимость создания регистра.

## ЦЕЛИ И ЗАДАЧИ ПРОЕКТА

Цель: создание единой национальной базы данных пациентов с гипопаратиреозом на территории Российской Федерации.

Задачи регистра:

1. оценка эпидемиологических показателей (распространенность, заболеваемость, летальность, смертность) у пациентов с гипопаратиреозом в РФ;

2. сбор и анализ информации о течении заболевания, включая:

Результаты анализа базы данных регистра позволят оптимизировать ведение пациентов с данной патологией, в том числе будут важны для разработки и актуализации клинических рекомендаций и улучшения качества оказания медицинской помощи.

## МАТЕРИАЛЫ И МЕТОДЫ

Всероссийский регистр гипопаратиреоза создан на основе электронной базы данных отделения патологии околощитовидных желез ФГБУ «НМИЦ эндокринологии» Минздрава России, включившей пациентов с верифицированным диагнозом за период 2017–2020 гг. С 2020 г. база данных трансформирована в электронную информационно-аналитическую платформу с единой картой регистра на всей территории РФ с онлайн-вводом данных и динамической системой аналитики (вход на платформу: http://diaregistry.ru/).

На 01.07.2021 г. регистр включает 528 пациентов (509 взрослых и 19 детей) из 63 регионов РФ. В 8 регионах инициирован самостоятельный ввод пациентов (г. Москва, Московская, Магаданская, Белгородская, Тюменская области, Алтайский и Ставропольский край, Ямало-Ненецкий автономный округ).

## ЭТИЧЕСКИЙ КОМИТЕТ

Карта регистра гипопаратиреоза одобрена локальным этическим комитетом ФГБУ «НМИЦ эндокринологии» Минздрава России (протокол №18 заседания Комитета от 11.10.2017 г.). Обязательным условием является подписание информированного согласия на добровольное участие пациента в регистре и обработку личных данных.

## МЕТОДОЛОГИЯ ВЕДЕНИЯ РЕГИСТРА

Включению в регистр подлежат пациенты с хроническим послеоперационным и нехирургическим гипопаратиреозом любой этиологии (за исключением транзиторной и функциональной формы), без ограничений по возрасту и полу.

## КОДЫ ПО МКБ-10

E89.2 Гипопаратиреоз, возникший после медицинских процедур.

E20.0 Идиопатический гипопаратиреоз.

E20.8 Другие формы гипопаратиреоза*.

E20.9 Гипопаратиреоз неуточненный*.

* При указании данных кодов по МКБ-10 необходима расшифровка диагноза.

В регистр гипопаратиреоза не вносятся данные о пациентах с обратимыми формами заболевания, такими как:

1. функциональный гипопаратиреоз, развивающийся в результате нарушения обмена магния и характеризующийся восстановлением функции ОЩЖ после коррекции гипо-/гипермагниемии;

2. транзиторный послеоперационный гипопаратиреоз, при котором восстановление функции ОЩЖ происходит, как правило, в течение первых 4–6 нед после операции на органах шеи.

Критерии выбывания/снятия с учета:

1. отзыв письменного добровольного информированного согласия пациента.

В регистр вносится информация о пациентах, обратившихся по поводу данного заболевания за амбулаторной помощью или госпитализированных в стационар лечебного учреждения субъекта РФ.

Периодичность внесения данных в регистр определяется клиническим статусом конкретного пациента, необходимой частотой обследования и коррекцией терапии — в среднем от 1 до 3–4 визитов в год.

## СТРУКТУРА КАРТЫ РЕГИСТРА

Карта пациента представляет собой форму для заполнения основных данных и включает в себя:

1. паспортную часть (ФИО, дата рождения, регион проживания и др.);

2. диагноз (послеоперационный, аутоиммунный, другие формы наследственного гипопаратиреоза, идиопатический и прочие формы заболевания);

3. сведения на текущий момент (вносятся системой автоматически из последнего заполненного визита пациента);

4. визиты пациента — «полный» и «динамический»;

5. лечение (заполняется системой автоматически и отображает схему лечения из последнего заполненного визита пациента);

6. жизненный статус пациента (жив/умер/нет данных).

При первичном вводе данных заполняются: паспортная часть, клинический диагноз и «полный визит» пациента. При последующем мониторинге заполняется только раздел визита, и в данном случае может быть выбрано внесение как «полной», так и «динамической» его формы.

«Полная» форма визита пациента включает:

-острые осложнения (частота госпитализаций с острой гипокальциемией);

-данные о наличии патологии почек, центральной нервной системы, органов зрения, желудочно-кишечного тракта, сердечно-сосудистой и костно-мышечной систем;

-основное лечение: препараты активной формы витамина D (альфакальцидол/кальцитриол) и соли кальция;

-дополнительная терапия: колекальциферол, тиазидные диуретики, препараты магния и другие;

-сопутствующая терапия, оказывающая влияние на состояние фосфорно-кальциевого обмена (в формате отметки «да/нет»);

«Динамическая» форма визита — краткий вариант, который состоит только из укороченной версии лабораторного блока, лечения пациента и статуса заболевания.

## СИСТЕМА ПОДДЕРЖКИ ПРИНЯТИЯ РЕШЕНИЙ

Система поддержки принятия решений (СППР) (Clinical decision support system (CDSS)) — аналитическая опция системы, предназначенная для помощи врачам и иным медицинским специалистам в работе с задачами, связанными с принятием клинических решений.

Рабочее определение было предложено Робертом Хейвордом (Robert Hayward), сотрудником Центра доказательной медицины (Centre for Health Evidence): «Системы поддержки принятия врачебных решений связывают результаты клинических исследований с данными, имеющимися в отношении конкретного пациента, влияя на выбор врачебного решения для более эффективного оказания медицинской помощи».

Разработка и внедрение СППР относятся к важнейшему современному направлению развития искусственного интеллекта в медицине.

Основной принцип СППР — непрерывный мониторинг и анализ поступающих данных с формированием уведомлений о возникшей клинической ситуации, выходящей за «рамки нормативных значений и показателей» и выведением этих уведомлений на экраны мониторов рабочих мест специалистов [7].

Реализованная в регистре гипопаратиреоза СППР основана на позициях, утвержденных клиническими рекомендациями (2021 г., https://cr.minzdrav.gov.ru/recomend/627_2), и призвана оказать поддержку посредством выведения алгоритма о необходимости дополнительного обследования и/или коррекции терапии, исходя из заполненных лабораторных данных пациента и указанной терапии, проанализированных системой.

Основными задачами СППР являются привлечение внимания врача-специалиста к конкретной клинической ситуации в состоянии пациента, свидетельствующей о нарушении лабораторных показателей или наличии признаков неэффективности проводимой терапии, а также визуализация алгоритма его возможных действий в соответствии со стандартом — при этом клиническое решение в каждом конкретном случае, безусловно, принимается непосредственно лечащим врачом-эндокринологом.

В предлагаемом алгоритме СППР анализируются показатели фосфорно-кальциевого обмена, их отклонение от референсного диапазона (внесенного специалистом или преднастроенного**, если данные не внесены), на основании чего формируются «подсказки» по коррекции терапии и дообследованию.

** Преднастроенные границы референсного диапазона установлены в соответствии с клиническими рекомендациями по гипопаратиреозу и применяются в случае, если не внесены значения минимальных и максимальных границ референсного диапазона при заполнении визитов пациента.

Пример.

Если показатели общего кальция, фосфора и кальция в суточной моче соответствуют целевому диапазону — всплывающее окно сообщит о наличии у пациента лабораторной компенсации заболевания и предложит необходимую кратность обследования в динамике и его перечень.

В случае если внесенные показатели выходят за границы референсного диапазона, СППР на основании указанной лекарственной терапии предложит вариант ее коррекции (рис. 1).

**Figure:**
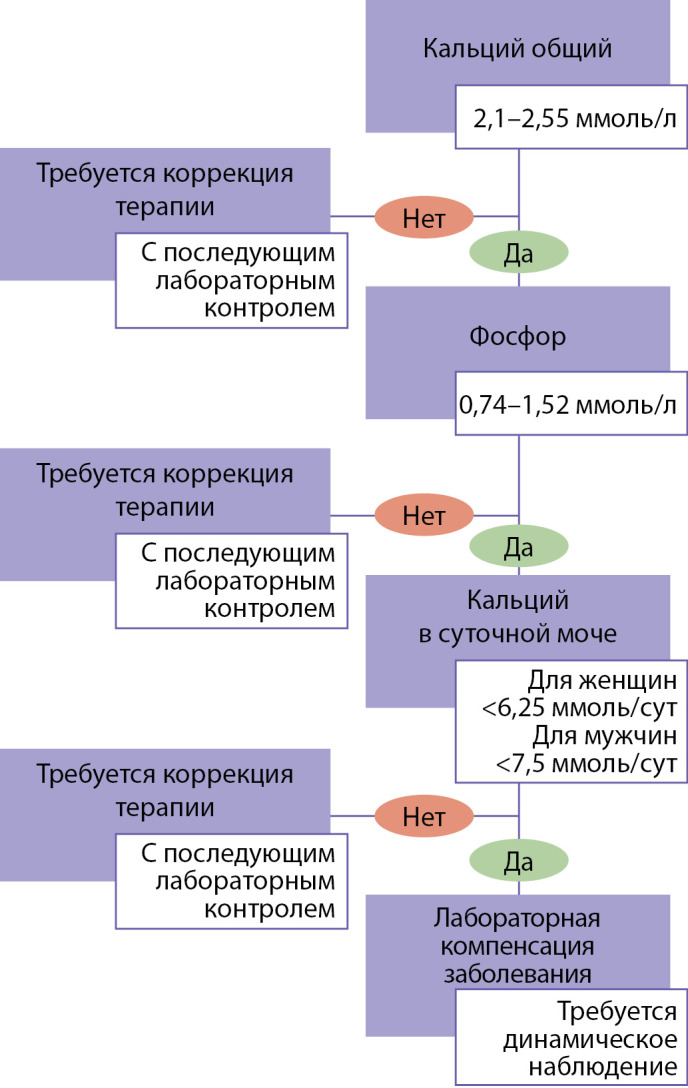
Рисунок 1. Блок-схема алгоритма системы поддержки принятия решений (СППР).

В алгоритм СППР также вложены «подсказки» по установлению статуса заболевания — компенсация/субкомпенсация/декомпенсация. Так, лабораторная компенсация заболевания предусматривает целевые показатели по основным лабораторным параметрам — кальцию, фосфору крови и кальцию суточной мочи. При наличии отклонения одного или всех ключевых показателей СППР будет обращать внимание специалиста на возможное наличие суб- или декомпенсации заболевания.

СППР призвана также оценивать полноту внесения данных в регистр, и в этом качестве система будет выдавать напоминание о необходимости дообследования пациента, если один или несколько ключевых показателей фосфорно-кальциевого обмена не заполнены.

## ЗАКЛЮЧЕНИЕ

В связи с высокой социальной значимостью гипопаратиреоза, обусловленной хроническим течением заболевания, требующим пожизненной многокомпонентной терапии, риском развития множественных осложнений и инвалидизации в трудоспособном возрасте, создан Всероссийский регистр гипопаратиреоза, который позиционируется в качестве высокофункциональной информационно-аналитической платформы с широкими возможностями анализа и планирования, эффективного инструмента клинико-эпидемиологического мониторинга данной патологии в масштабах всей страны. Дополнительные возможности открывает новая аналитическая функция регистра, позволяющая внедрять современные СППР.

Развитие регистра пациентов с гипопаратиреозом позволит улучшить качество оказания медицинской помощи этим пациентам, в том числе осуществлять своевременную диагностику, оптимизировать схемы лечения и снизить риски развития осложнений. Всероссийский регистр гипопаратиреоза также призван выступать в качестве ценной научной базы для планирования и реализации новейших разработок по лечению данного заболевания.

Регистр гипопаратиреоза расположен на единой платформе регистров эндокринопатий ФГБУ «НМИЦ эндокринологии» Минздрава России (http://gipopt.clin-reg.ru/). Помимо рабочего портала, на странице регистра существуют информационные разделы, в том числе ссылки на статьи, клинические рекомендации, расписание школ для специалистов; указана контактная информация, а также вывешена форма информированного добровольного согласия пациента на включение в регистр.
